# Investigation on Hot-Carrier Injection Mechanisms and Reliability Design-Based Strategies in Cryogenically Operated CMOS ROICs for Infrared Focal Plane Arrays

**DOI:** 10.3390/s26196254

**Published:** 2026-10-02

**Authors:** Ayoub El Abdi, Philippe Martin-Gonthier, Valentin Badefort-Baril

**Affiliations:** CIMI Integrated Image Sensor Laboratory, Institut Supérieur de l’Aéronautique et de l’Espace (ISAE-SUPAERO), Université de Toulouse, 31055 Toulouse, France; philippe.martin_gonthier@isae-supaero.fr (P.M.-G.); valentin.badefort-baril2@isae-supaero.fr (V.B.-B.)

**Keywords:** index terms—ROICs, CMOS, infrared sensors, FPA, hot-carrier injection, reliability, cryogenic temperatures, MOSFET

## Abstract

Cryogenic operation of CMOS read-out integrated circuits (ROICs) is essential for high-performance infrared focal plane arrays but significantly affects carrier transport and device reliability. This work investigates hot-carrier injection (HCI) mechanisms in 180 nm NMOS transistors operating from room temperature down to 140 K. Electrical characterization and accelerated stress measurements reveal a strong impact of hot-carrier effects at low temperatures, resulting in increased degradation of device characteristics, particularly under channel hot-electron (CHE) and drain avalanche hot carrier (DAHC) stress conditions. Time-to-failure modeling confirms accelerated degradation kinetics while validating conventional substrate-current-based reliability methodologies at cryogenic temperatures. Geometry-based design strategies, notably gate-length optimization, are proposed to mitigate cryogenic HCI effects, enabling an improved performance–reliability trade-off. These results provide key guidelines for design reliability of cryogenic CMOS ROICs for infrared imaging applications.

## 1. Introduction

Infrared (IR) detectors are fundamental components in a wide range of scientific, spatial, and defense applications, notably for Earth observation, target tracking and environmental monitoring. Their performance relies critically on both the photosensitive material and the associated Read-Out Integrated Circuit (ROIC), which is commonly implemented using Complementary Metal Oxide Semiconductor (CMOS) technology. To improve the performance of the IR-sensitive layers, detectors are usually operated at cryogenic temperatures: around 200 K for SWIR, 140 K for MWIR, and 80 K (and lower) for LWIR. Cooling the detector significantly reduces thermal generation and leakage currents within the sensing material, minimizing dark current and thermal noise. As the ROIC and IR-sensitive layer are hybridized to create the Focal Plane Array (FPA), by extension, the ROIC is also operated at cryogenic temperatures.

However, operating CMOS-based ROICs under cryogenic conditions introduces substantial changes in the fundamental physical mechanisms governing device operation. For example, the phonon population is markedly reduced, leading to a decrease in carrier–phonon scattering and consequently leading to an increase in carrier mobility [1]. Simultaneously, the decrease in intrinsic carrier concentration increases the magnitude of the bulk Fermi potential, requiring a larger surface potential to reach strong inversion and consequently increasing the threshold voltage. Incomplete dopant ionization may additionally modify the free-carrier concentration and charge distribution, depending on temperature, doping, and bias conditions [1,2]. Additionally, the semiconductor bandgap widens as temperature decreases, modifying the intrinsic carrier density and shifting the threshold voltage of MOS transistors [1]. The trapping and detrapping dynamics of charge carriers at the dielectric interface and oxide trap sites are also affected: their rates slow down significantly, resulting in a change in the temporal response and noise characteristics of the devices [1]. Collectively, these cryogenically induced phenomena lead to observable variations in the transistor’s static and dynamic parameters, including threshold voltage, subthreshold slope, leakage current, transconductance.

One of the most critical concerns of HCI in CMOS design is reliability. HCI phenomenon also affects low-frequency noise [3] and electroluminescence [4]. Photons emitted by hot carriers may generate parasitic signals in FPA pixels, causing background glow and optical crosstalk. Blooming may also occur if these signals induce saturation and charge spreading. Although hot-carrier injection (HCI) mechanisms are well understood at room temperature, their behavior and impact under cryogenic conditions remain insufficiently investigated. This limited understanding of how low temperatures affect injection mechanisms poses a significant challenge when addressing HCI-related reliability issues in the design of CMOS ROICs for infrared detectors. Developing a deeper insight into the temperature-dependent evolution of HCI and establishing design methodologies that explicitly integrate these reliability considerations is therefore essential to achieve an optimal balance between performance and long-term durability in cryogenic CMOS ROICs. Although CMOS ROICs incorporate both NMOS and PMOS devices, this study focuses on NMOS hot-carrier reliability. Previous cryogenic studies report greater PMOS resistance to HCI [5] and stronger cryogenic acceleration of NMOS degradation [6]. These findings motivate the NMOS focus without implying negligible PMOS degradation.

The aim of this paper is to investigate hot-carrier injection mechanisms at cryogenic temperatures and to use the results to develop design methodologies that explicitly incorporate temperature-dependent reliability considerations. Section 2 reviews the main physical mechanisms leading to HCI. Section 3 presents the material and characterization setup used for this work.

In Section 4, the impacts of the different operating points and temperatures are evaluated using a common indicator of HCI, substrate current which allows to study device reliability and time-to-failure modeling for cryogenic temperatures. Finally, Section 5 concludes the paper and outlines key perspectives.

## 2. HCI Main Physical Mechanisms

Hot carrier injection (HCI) in MOSFET occurs when charge carriers gain sufficient kinetic energy from high lateral electric fields near the drain region. These energetic carriers can surmount the Si–SiO_2_ potential barrier or generate interface traps and become trapped within the gate oxide or at the generated interface traps, inducing permanent damage. Over time, this process manifests as a shift in threshold voltage, degradation of transconductance and subthreshold slope [7], and an increase in interface trap density, implying an increase in random telegraph signal (RTS) noise [3]. Moreover, electroluminescence phenomena resulting from radiative recombination of energetic carriers can be observed under strong stress conditions, providing additional insights into the degradation mechanisms [4].

Several hot-carrier injection modes exist, each governed by different physical mechanisms and bias conditions; they are listed below and shown in Figure 1.

- Channel Hot Hole (CHH) injection: In the linear region (VDS < < VGS − VT), the lateral electric field within the channel is relatively weak. However, the substantial potential difference between the gate and drain can generate a significant vertical electric field. This condition may promote CHH injection, contributing to the generation of interface states and gradual degradation of the S_i_/S_i_O_2_ interface [8,9,10].
- Drain avalanche hot carrier (DAHC) injection: In the saturation regime around V_GS_ ≈ V_DS_/2 and for high VDS values, the strong lateral electric field near the drain junction generates energetic electrons and holes by impact ionization. This process results in substrate current and represents the most critical degradation mechanism at room temperature, as both hot electrons and hot holes contribute to the generation of the interface states [11,12].
- Channel Hot Electron (CHE) injection: As V_GS_ is further increased around VDS ≈ VGS, the electric field at the drain junction is reduced, leading to a decrease in the substrate current. Conversely, the enhanced vertical electric field favors channel hot-electron injection through the escape of so-called “lucky” electrons, resulting in a significant degradation of the gate oxide and the S_i_O_2_/S_i_ interface, particularly at low temperatures (77 K) [13].
- Substrate Generated Hot Electron (SGHE) injection: under strong vertical electric fields, typically induced by high substrate bias, SGHE injection originating from minority carriers generated by secondary impact ionization or bremsstrahlung radiation [14,15] becomes more important in ultra-scaled MOS devices.
- In modern transistors with ultra-thin gate oxides, Fowler–Nordheim and direct tunneling mechanisms can also play a significant role in hot-carrier injection phenomena [16].

## 3. Material and Characterization Setup Description

The devices under test (DUT) investigated here are standard 3.3 V n-channel MOSFETs from a commercially available 180 nm CMOS technology node with channel width-to-length ratios of W/L = 10/0.34 and 10/0.5. The transistors are built on a 5 Ω·cm p-type epitaxial silicon substrate, with a gate oxide thickness of 7 nm. These transistors are arranged in a test structure designed for characterization with a cryo-prober.

The transistors are subjected to accelerated DC hot-carrier stress following the measurement procedure described in JESD28-A [17]. Stress conditions near maximum substrate current target the DAHC regime, whose worst-case relevance is experimentally assessed across the investigated temperatures in Section 4.2. The complementary addendum JESD28-1 [18] provides methods for analyzing the resulting degradation data and extracting time to failure (TTF). The monitored device parameters are permanently recorded after each stress interval, and the test is continued until the failure criterion Y_tar_ is met, defined in this work as a 10% deviation from the initial parameter value. The substrate/drain current ratio analysis method is used to predict the time for the chosen parameter to reach the degradation criterion under use-bias conditions at room and cryogenic temperatures. The time to failure model is given by (1):

(1) $$\mathrm{TTF}.I_{D,\mathrm{stress}}=\mathrm{HW}{\left(\frac{I_{\mathrm{sub},\mathrm{stress}}}{I_{D,\mathrm{stress}}}\right)}^{-m}$$

(2) with TTF=YtarC1n

where TTF is the time to reach the specified failure criterion, W is the transistor width, I_sub,stress_ and I_D,stress_ are the substrate and drain currents, respectively, under the MOSFET stress bias conditions V_DS,stress_ and V_GS,stress_. C, n, m, and H are the fitting parameters.

## 4. Measurement Results and Discussion

### 4.1. HCI Monitoring

In this section, we study the effect of the temperature on the substrate current. Based on experimental data and models from the literature, the key parameters that depend on the temperature are determined, and their impact on the degradation of interface states is evaluated.

The substrate current, shown in Figure 2, is commonly used to monitor HCI-induced degradation in NMOS devices, particularly under the bias condition V_GS_ ≈ V_DS_/2, which is commonly regarded as the worst-case stress condition, as it maximizes the concurrent contribution of hot electrons and hot holes to the degradation of the silicon/oxide interface.

The substrate current, which originates from impact ionization by the maximum electric field E_m_ near the drain, is given by [19]:

(3) $$I_{\mathrm{sub}}=C_{1}.I_{D}.\exp\left(-\frac{\phi_{i}}{q\lambda_{e}E_{m}}\right)$$

where ϕ_i_ is the impact ionization energy, λ_e_ is the mean free path for electrons, q is the elementary charge, and C_1_ is a constant to be determined experimentally.

The maximum electric field can be expressed by a semi-quantitative analytical model E_m_ = (V_DS_ − V_DSAT_)/χ, where V_DSAT_ is the drain saturation voltage and χ is a specific length of the transistor [19], so (3) can be rewritten as

(4) $$\frac{I_{\mathrm{sub}}}{I_{D}}=C_{1}.\exp\left[-\frac{\chi .\phi_{i}}{q.\lambda_{e}.\left(V_{\mathrm{DS}}-V_{\mathrm{DSAT}}\right)}\right]$$

This expression is verified by plotting I_sub_⁄I_D_ as a function of 1/(V_DS_ − V_DSAT_) at room temperature and the results are shown in Figure 3. The model proves to be accurate for (V_DS_ − V_DSAT_) ≥ 2.3 V (left side). At smaller excess drain voltages, corresponding to larger inverse-voltage coordinates, the measurements deviate from the extrapolated model. This deviation is consistent with the limitations of the simplified electric-field description (right side), which does not explicitly resolve the spatial field profile or carrier energy distribution governing impact ionization. The dashed portion of the model line indicates extrapolation outside the identified agreement range.

From the fitted line in Figure 3, C_1_ = 1.95 is obtained, which is close to 1.93 [9] and two [19], and the slope S = χ.ϕ_i_ ⁄ q.λ_e_ = 17 V. Heremans et al. [9] extended this analysis to cryogenic temperatures and demonstrated that C1 is almost independent of temperature, assuming that χ is independent of the temperature, which is reasonable since from [19].

(5) $$\chi \approx \sqrt{\frac{\varepsilon_{\mathrm{si}}}{\varepsilon_{\mathrm{ox}}}.T_{\mathrm{ox}}.\chi_{j}^{\prime }}$$

One can obtain $\lambda_{e,h}^{T1\;K}/\lambda_{e,h}^{T2\;K}$ for any desired temperature where (4) is validated. Here χ_j_’ is related to the junction depth, T_ox_ is the oxide thickness, and ε_si_ and ε_ox_ are the dielectric constants of silicon and the dioxide silicon, respectively.

To extend the analysis over the full temperature range, the temperature dependence of the parameters governing the substrate current must be considered. However, the dominant temperature effect on hot-carrier behavior is traditionally attributed to variations in the carrier mean free paths λ_e_ and λ_h_. A widely accepted expression introduced by Crowell and Sze [20] describes this dependence as:

*λ*(*T*) = *λ*_0_*tanh*(*E_R_*/2*kT*) (6)

where E_R_ = 63 meV is the optical phonon energy in silicon. This relation indicates that the mean free path decreases with increasing temperature. Although this temperature dependence is expected to weaken with aggressive device scaling [21], it remains relevant for the technological node investigated in this work.

Another important temperature-dependent parameter is the drain saturation voltage V_DSAT_ (T), and it can be expressed by [22]:

(7) $$V_{\mathrm{DSAT}}=\frac{2\left(V_{\mathrm{GS}}-V_{T}\right)/m}{1+\sqrt{1+\frac{2\left(V_{\mathrm{GS}}-V_{T}\right)}{mF_{C}\left(L-L_{s}\right)}}}$$

where F_C_ is the critical field for velocity saturation, L is the effective gate length, L_s_ is the length of the velocity saturation region, m is the body effect coefficient, and V_T_ is the threshold voltage.

V_DSAT_ (T) variation is mainly driven by the temperature dependence of the critical electric field F_C_ and the threshold voltage V_T_, assuming a weak temperature dependence of the body effect coefficient m. The critical field is given by [21]:

(8) $$F_{C}\left(T\right)=\frac{v_{\mathrm{sat}}\left(T\right)}{\mu_{\mathrm{eff}}\left(T\right)}$$

A decrease in temperature leads to an increase in carrier mobility μ_eff_ and V_T_, along with a reduction in the critical electric field F_C_, resulting in a lower drain saturation voltage V_DSAT_. This enhances the peak electric field near the drain E_m_, thereby increasing impact ionization and consequently the substrate current I_sub_, and the fraction of hot carriers relative to the total channel carrier population [9].

From these expressions, the substrate current, and thus the degradation of device characteristics, is governed by variations in the effective gate length L, the junction depth, the oxide χ_j_’ thickness T_ox_, the biasing conditions, and the operating temperature through their effects on the carrier mean free path λ and the maximum lateral electric filed in the channel E_m_. They are therefore employed to analyze NMOS devices degradation at both room and cryogenic temperature and to develop design strategies to mitigate HCI-induced degradation.

### 4.2. Device Reliability

At room temperature, the bias condition corresponding to the maximum substrate current is commonly regarded as the worst-case stress condition for long-channel NMOS devices. This condition is typically associated with V_GS_ ≈ V_DS_/2, where impact ionization and interface-state generation are maximized. However, this assumption does not necessarily hold for short-channel devices, where quasi-ballistic transport and velocity overshoot effects become significant and modify the carrier heating dynamics [21,23]. Moreover, the worst-case stress condition is expected to depend on temperature, particularly under cryogenic operation [11]. For these reasons, the validity of the conventional worst-case bias condition must be revisited. In ROIC design, electrical parameters such as gm, Vth, and Ion govern key performance characteristics, and their shifts indicate degradation. Here, we selected the linear threshold-voltage V_T,lin_ shift as the primary indicator for comparing degradation across stress conditions, temperatures, and channel lengths.

To this end, separate NMOS devices with W/L = 10/0.34 are stressed at 295 K, 190 K, and 140 K under a fixed high V_DS,stress_ and five selected V_GS,stress_ values, each applied for a cumulative stress duration of 10^4^ s, as depicted in Figure 4. At the end of each stress condition, the degradation behavior is evaluated by extracting the linear threshold voltage shift V_T,lin_, derived from the maximum transconductance g_m(lin,max)_ of the I_D_-V_GS_ curve with V_DS_ = V_DS(lin)_ = 50 mV, V_BS_ = 0 V. This approach enables the activation of different HCI regimes, including DAHC, CHE, and CHH injection modes.

The results clearly indicate that ΔV_T_ degradation is more severe at cryogenic temperatures across all injection regimes. In particular, for the DAHC injection mode, where the generation of interface states dominates, the threshold voltage shift is more than twice as large at 190 K and reaches up to 3.5 times the room-temperature value at 140 K. It is important to note that this enhanced ΔV_T_ degradation at cryogenic temperatures does not imply a higher density of interface states than at room temperature room temperature. Charge-pumping technique-based studies [9], which directly probe the density of fast interface states, have demonstrated that the activation energy for interface states generation is positive with respect to temperature. Consequently, for identical stress conditions, a larger number of interface states is generated at room temperature than at cryogenic temperatures. The pronounced threshold voltage shift observed at low temperatures is instead attributed essentially to the increased sensitivity of transistor characteristics to charged interface states and fixed oxide charges. In particular, the potential barrier at the source side of the degraded device has a stronger influence on carrier transport at cryogenic temperatures, such that an equivalent amount of interface states or fixed oxide charges induces a larger perturbation of the I–V characteristics than at room temperature, as reported in [9,13]. Additional contributions may arise from enhanced electron trapping at cryogenic temperatures, especially in shallow interface traps, due to reduced thermal emission rates. However, previous studies have shown that this effect alone is insufficient to account for the large threshold voltage shifts observed at low temperatures. Another plausible explanation involves the temperature dependence of the capture cross section of oxide traps, which has been reported to increase as temperature decreases [24,25], further amplifying the electrical impact of trapped charges at cryogenic temperatures.

For the CHE injection mode, a modest exacerbation in threshold voltage degradation is observed at 190 K, whereas at 140 K the CHE-induced ΔV_T(lin)_ increases by nearly an order of magnitude. This behavior is consistent with the enhanced electron injection probability at low temperatures. The reduced energy relaxation at cryogenic temperatures allows electrons to retain higher kinetic energies, increasing their ability to surmount the Si/SiO_2_ energy barrier and become trapped in the oxide. The same physical arguments invoked for DAHC injection therefore apply to the CHE regime: enhanced injection efficiency combined with the stronger electrostatic impact of trapped charges at low temperatures leads to a substantial increase in threshold voltage degradation. It has been reported in the literature that CHE injection can become the dominant degradation mechanism at temperatures above 80 K [13], a temperature range of particular relevance for long-wavelength infrared (LWIR) applications. In this study, no direct evidence of CHH injection was observed, as no negative threshold voltage shift was detected. This behavior is attributed either to; (1) an insufficient drain-to-gate potential to enable hole injection. Drain voltages up to 5.75 V were applied without measurable hole current, beyond which oxide breakdown occurred, preventing further investigation of this regime; or (2) CHH injection may still occur, but its effect can be masked by dominant electron trapping in the generated interface states, resulting in a net positive shift in Vth_,lin_ and concealing hole trapping in the gate oxide. To elucidate the role of CHH injection, advanced techniques such as charge pumping and floating-gate methods should be employed to directly assess interface-state generation due to CHH injection [9] and detect CHH-induced gate currents down to 0.1 fA [10].

These findings underscore the critical importance of accounting for temperature-dependent hot-carrier effects when assessing NMOS reliability under cryogenic operating conditions.

### 4.3. TTF Modeling and Design Trade-Off

In this part, the analysis is focused on the TTF behavior of the investigated NMOS devices. As demonstrated in IV.B, for the considered technology node and a minimum gate length of 0.34 µm, the maximum substrate current bias condition remains the worst-case stress condition, at least down to 140 K. This result confirms that reliability assessment methodologies based on the maximum substrate current criterion remain valid under these operating conditions, and that standard approaches such as [17] can be safely applied.

The TTF definition used throughout this work follows that introduced in Section 3. TTF modeling of the linear threshold voltage degradation is performed at 295 K, 190 K, and 140 K for the minimum gate length of 0.34 µm of the investigated technology node, as illustrated in Figure 5.

An important outcome is that the fitting exponent m remains nearly independent of temperature, which indicates that under these conditions, the main origin of the degradation of device characteristics is still the generation of interface states, and the critical energies for electrons and holes to form fast interface traps upon injection remain unaffected by temperature variations [9,13,19]. Consequently, within the proposed model, the only parameter exhibiting a clear temperature dependence is the fitting factor H, whose value decreases as temperature is reduced.

As discussed in IV.A, substrate current reflects the influence of temperature, device geometry, and bias conditions on hot-carrier activity, providing a basis for identifying mitigation strategies. Oxide thickness t_ox_ and junction depth X_j_ are largely fixed by the selected process. At FPA level, reducing V_DD_ can lower transistor voltage stress, but can also reduce the voltage available to maintain the required operating conditions and accommodate signal variations. Depending on the readout architecture, this may limit signal swing and charge-handling capacity, reduce dynamic range, or constrain readout speed. Its reliability benefit must therefore be weighed against the performance requirements of the target ROIC.

We therefore focus on increasing gate length L, an accessible design variable that reduces the lateral electric field while retaining the fabrication process and nominal supply voltage. Its costs in area, drive current, and capacitance must be balanced against the reliability benefit. Selective application to transistors exposed to significant HCI stress can limit its impact on the overall ROIC layout. As shown in Figure 2, the substrate current increases by factors of approximately 1.4 and 1.7 at 190 K and 140 K, respectively, compared to room temperature. In contrast, Figure 6 indicates that increasing the gate length from 0.34 µm to 0.5 µm and 1 µm reduces the substrate current by factors of 2.3 and 5.4, respectively. These results demonstrate that, for the present technology, the impact of device geometry on substrate current and hence on reliability is significantly stronger than that of temperature.

This trend is further confirmed by the TTF analysis presented in Figure 7, which shows TTF.I_D_ as a function of the substrate-to-drain current ratio Isub/I_D_.

Increasing the gate length effectively compensates the degradation exacerbation induced by cryogenic operation. Notably, devices with a gate length of 0.5 µm operated at cryogenic temperatures exhibit a longer lifetime than minimum-length devices with a gate length of 0.34 µm, even when the latter are operated at room temperature.

These results emphasize a critical performance–reliability trade-off. While shorter gate lengths are highly desirable to maximize drive current and transconductance, particularly for cryogenic applications, the associated increase in carrier energy and their enhanced impact at low temperatures significantly intensify hot-carrier effects, thereby degrading device reliability.

Conversely, moderate increases in gate length result in a substantial improvement in device lifetime, at the cost of only a limited performance penalty. This trade-off becomes especially critical for cryogenic electronics, where enhanced carrier transport further amplifies hot-carrier degradation mechanisms. Consequently, careful optimization of device geometry emerges as a key strategy to achieve an optimal balance between performance and reliability in low-temperature CMOS applications. The present TTF analysis is supported over 140–295 K; extrapolation to lower temperatures requires additional validation. For example, bulk freeze-out may increase substrate resistance and modify the local body potential [26], while band-tail states may affect carrier statistics and transport [27]. Temperature-dependent charge retention in shallow interface traps may also modify the electrical response [7]. These effects are not necessarily captured by a simple temperature-scaling factor, and their influence on degradation requires careful experimental investigation.

## 5. Conclusions and Perspectives

This work investigates HCI mechanisms and their impact on the reliability of NMOS transistors under cryogenic operation. Substrate currents is shown to be a reliable indicator of HCI, validating conventional worst-case stress conditions down to 140 K and also validating the standard reliability assessment methodologies [17]. The results demonstrate a significant exacerbation of HCI-induced degradation of device characteristics at low temperatures. Results also moderate gate-length scaling effectively optimizes the performance–reliability trade-off by reducing degradation with minimal performance penalty. However, the strong increase in CHE injection at 140 K indicates that alternative worst-case stress conditions may arise below this temperature.

## Figures and Tables

**Figure 1:**
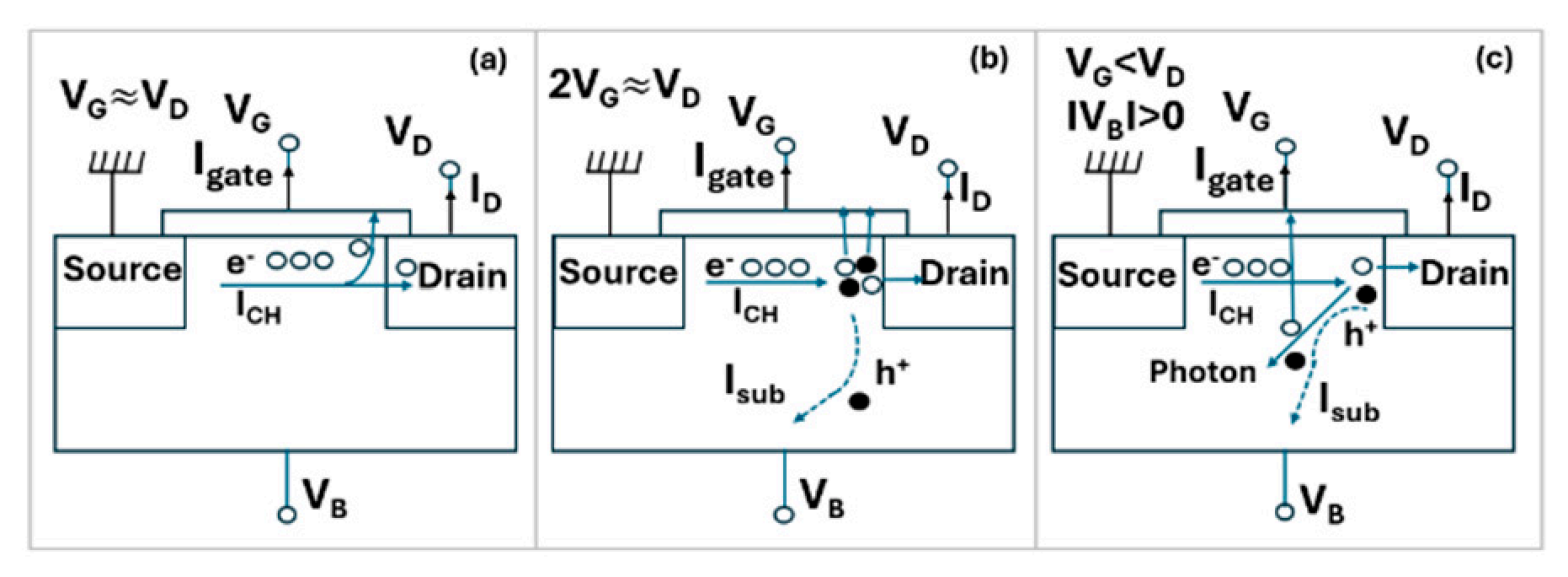
The main HCI mechanisms, (**a**) CHE, (**b**) DAHC, and (**c**) SHGE.

**Figure 2:**
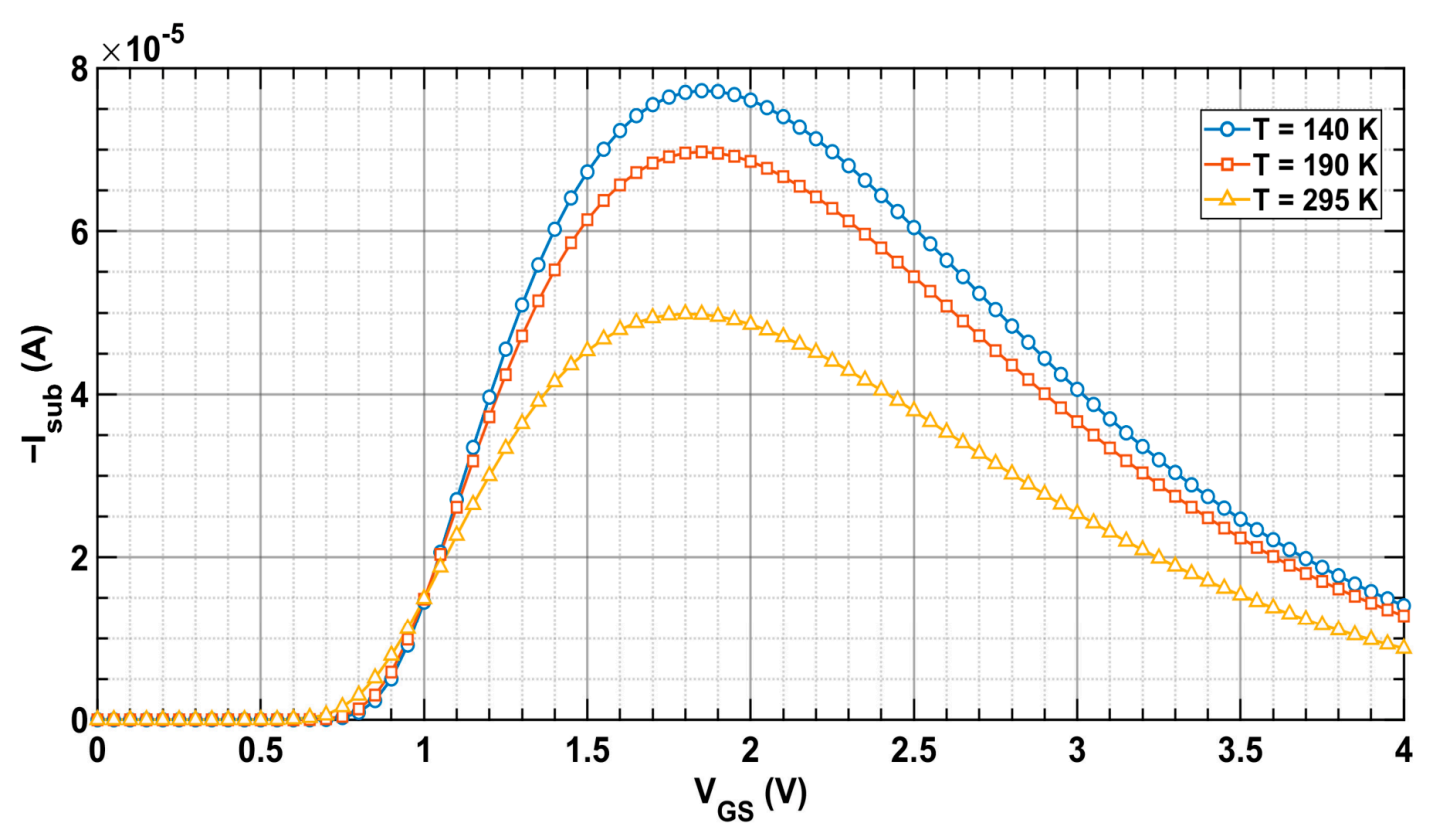
Substrate current measured at different temperatures for an NMOS transistor with W/L = 10/0.34, at V_DS_ = V_DD_ = 3.3 V.

**Figure 3:**
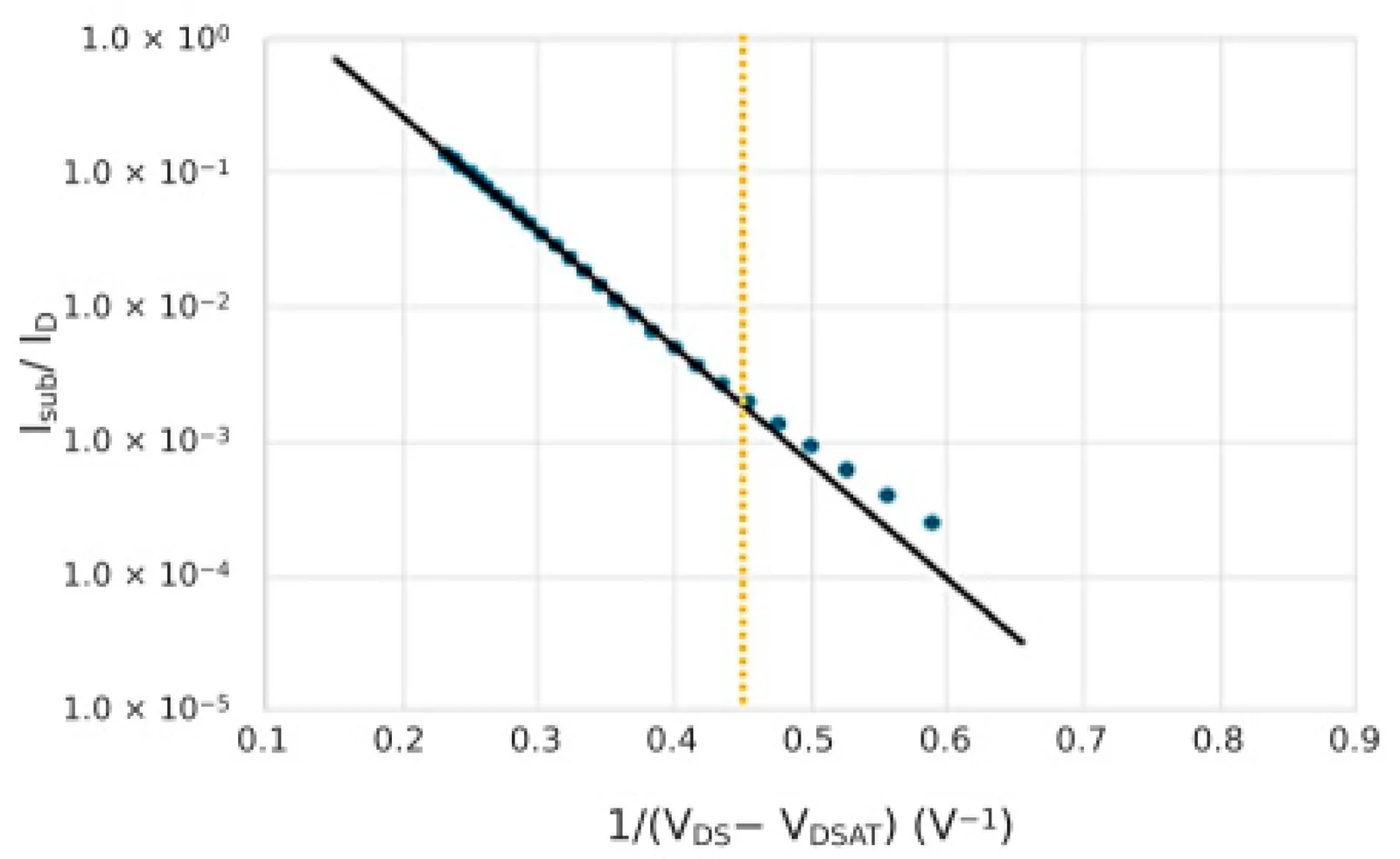
Ratio I_sub_/I_D_ versus 1/(V_DS_ − V_DSAT_) measured at several drain voltages for V_GS_ = 2.5 V at 295 K. The solid black line corresponds to the best fit of the measurement according to (4).

**Figure 4:**
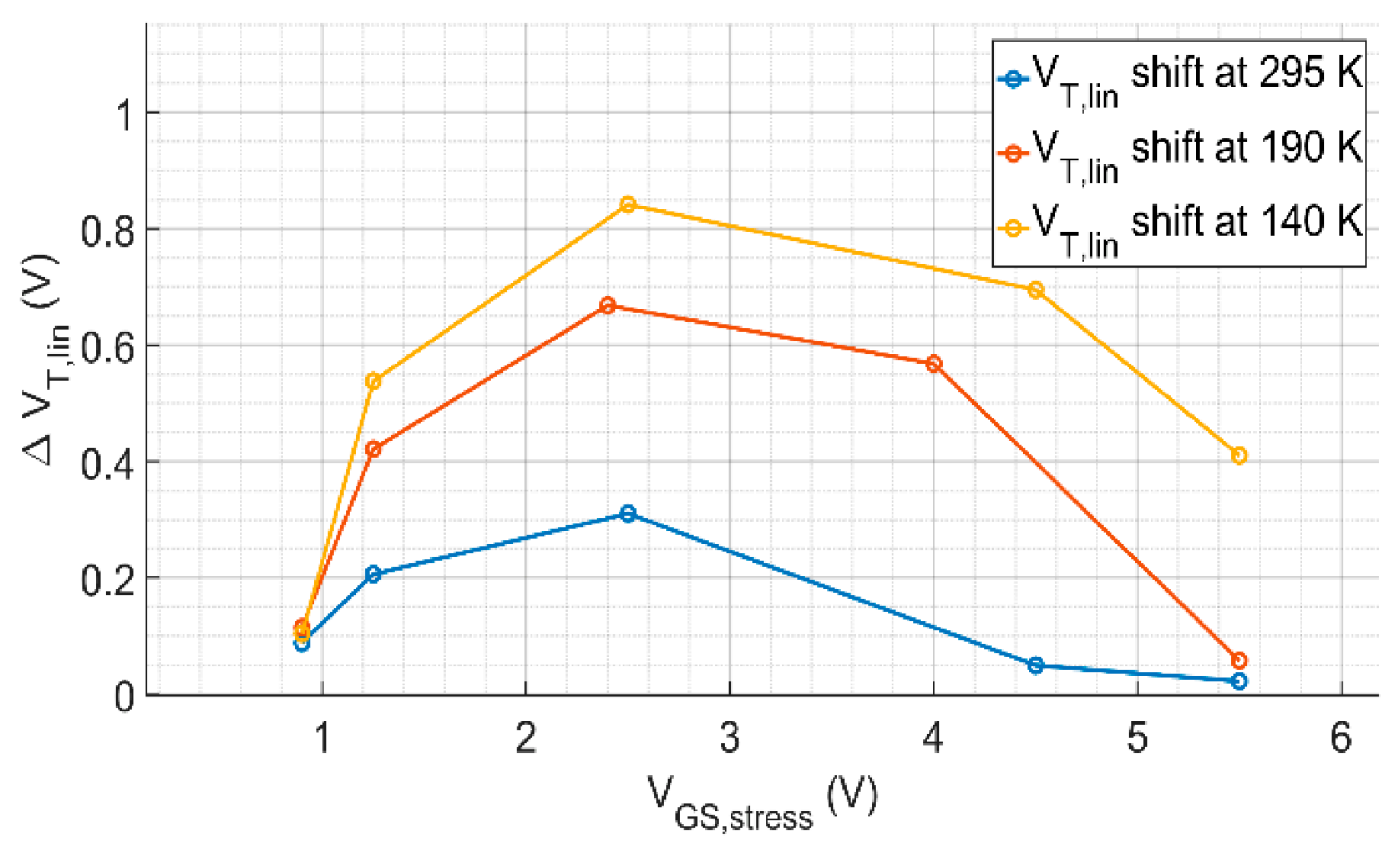
Linear threshold voltage degradation measured at 295 K, 190 K, and 140 K for NMOS transistors W/L = 10/0.34 under a drain stress voltage V_DS,stress_ = 5.5  and different gate stress voltages V_GS,stress_ applied for 10^4^  s.

**Figure 5:**
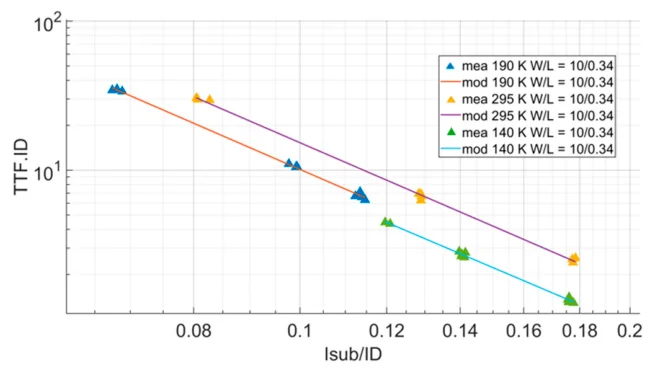
TTF models (solid lines) and measurements (symbols) for NMOS transistors with W/L = 10/0.34, measured at 295 K, 190 K, and 140 K.

**Figure 6:**
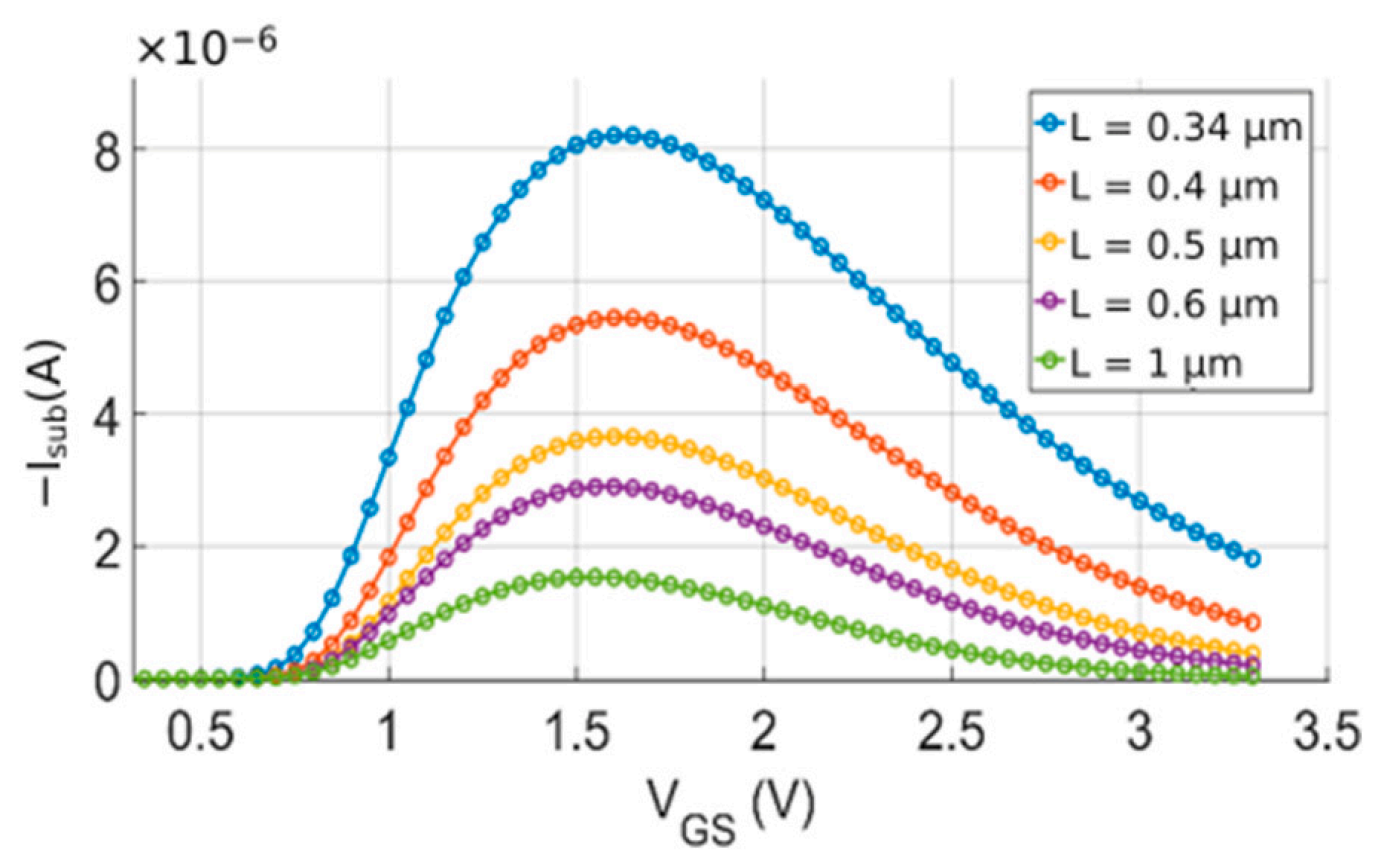
Substrate current as a function of gate voltage for different gate lengths, with W = 10 µm, for an NMOS transistor measured at 295 K and V_DS_ = V_DD_ = 3.3 V.

**Figure 7:**
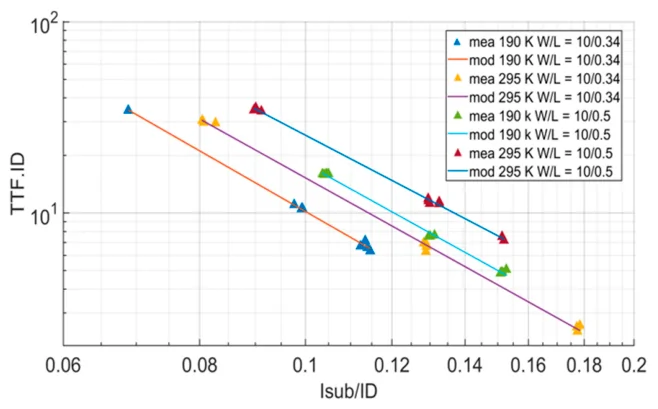
Time to failure models (solid lines) and measurements (symbols) for NMOS transistors with gate lengths of 0.34 µm and 0.5 µm, with W = 10 µm, measured at 295 K and 190 K.

## Data Availability

The data are available upon request.
